# Dual-polarization electromagnetic window simultaneously with extreme in-band angle-stability and out-of-band RCS reduction empowered by flip-coding metasurface

**DOI:** 10.1515/nanoph-2025-0386

**Published:** 2025-10-28

**Authors:** Heng-Yang Luo, Tie-Fu Li, Jia-Fu Wang, Yu-Xiang Jia, Rui-Chao Zhu, Xiao-Long Liang, Zhi-Hui Zhang, Min Zhou, Shao-Bo Qu

**Affiliations:** Shaanxi Key Laboratory of Artificially-Structured Functional Materials and Devices, 66488 Air Force Engineering University, 0053haanxi, 710051, China; Aerospace Metamaterials Laboratory of SuZhou Laboratory, Suzhou, Jiangsu 215004, China; Air Traffic Control and Navigation College, Air Force Engineering University, Xian, 710051, China

**Keywords:** integrating, longitudinally-coupled, dual-polarization angle stable EM window (0°–80°), radar cross-section (RCS) reduction

## Abstract

Achieving high electromagnetic (EM) wave transmission with excellent angular stability is crucial for communication, detection, and guidance but remains challenging, especially when integrating other functions like out-of-band radar cross-section (RCS) reduction, which often degrades transmission. In this work, we propose to solve this problem by utilizing the longitudinal design freedom of metasurface. To this end, a typical longitudinally-coupled structure is proposed as the meta-atom for designing metasurfaces, which is composed of one layer of metallic meshes and one layer of metallic patch. By leveraging the synergistic effect of the plasma oscillation of the metallic mesh and the Lorentz resonance effect of the metal patch within meta-atom, we obtain a dual-polarization angle stable EM window (0°–80°) within the operating band. On this basis, without altering the structural parameters of the meta-atom, we utilize the longitudinal dimension to encode the reflection phases of out-of-band EM waves by flipping the meta-atom structure longitudinally, which can integrate out-of-band radar cross-section (RCS) reduction function without affecting the in-band transmission performance. To demonstrate this idea, prototypes were designed, fabricated and measured. Fabricated prototypes show good agreement between measurements and simulations, validating the method. This opens new paths for multifunctional EM windows in next-gen communication and radar systems.

## 1 Introduction

Electromagnetic (EM) waves will be reflected at the two interfaces due to impedance mismatch, which leads to poor transmission performance, enhanced reflected signals, and further increases the reflection of radar waves, especially for two materials with a large difference in dielectric constant. To enhance the transmission performance, the antenna radome usually adopts the half-wave wall design method [1], [2], [3], [4]. However, the half-wave wall limits the thickness and the transmission efficiency drops sharply when the angle increases. For TM polarized waves, they have a Brewster angle that can increase the transmission effect, while this is not applicable to TE polarized waves [5], [6], [7]. Therefore, in practical application scenarios, in addition to meeting the requirement of good transmission effect, it is crucial to ensure the wide angle domain angular stability under the two polarization modes (TE polarization and TM polarization). Since its inception, the metasurface has demonstrated excellent electromagnetic regulation capabilities and has played a significant role in addressing the aforementioned challenges [8], [9], [10]. Meanwhile, the suppression of reflected wave signals also deserves key attention.

It is precisely due to the critical role of electromagnetic transmission enhancement technology and the impetus from urgent practical application demands that many researchers have conducted relevant studies. Yongzhi Li et al. achieved an enhancement of TE-polarized transmittance by loading long metal strips in the fiber antireflection ceramic material plate [11]. Yuchu He et al. designed a double-layered fishnet antireflection structure which achieves excellent antireflection effects for both TE and TM polarizations [12]. Fengyuan Yang et al. designed a cascaded antireflection metamaterial which achieves high transmission of EM waves from air to water within the target band [13]. In order to expand polarization, Nima Bayat-Makou et al. designed a dual-polarization antireflection structure which can adjust its structural parameters according to the wave impedance of the medium within the target band to achieve efficient transmission of EM waves [14]. With the deepening development of information warfare, it is not only essential to achieve efficient electromagnetic transmission within the band but also crucial to consider stealth performance outside the band. Since its inception, metasurface technology has demonstrated an inherent capability to flexibly control EM waves [15], [16], [17], attributed to its exceptional electromagnetic properties.

It is already highly challenging to achieve EM waves transmission with exceptional angular stability. Furthermore, this capability plays a crucial and broad role in practical applications such as communication, detection, and guidance [18], [19]. If additional electromagnetic functionalities are integrated, it could potentially compromise the performance of EM waves transmission. Additionally, one of the most significant branches within the field of electromagnetic functions is radar cross section (RCS) reduction. The most basic way to reduce the RCS of a radome is to use the exterior design to reflect out-of-band EM waves away from the direction of the incoming waves [20], [21]. However, with the development of anti-stealth technology and the improvement of radar detection capabilities, relying solely on exterior design is no longer sufficient for radome to meet the survival conditions in complex EM environments. Later, researchers developed a method to reduce the RCS of the radome by loading absorbing materials [22], [23], [24], [25], while this method would add extra weight, hindering its application in the aircraft field. In addition, the absorbing materials loaded would seriously affect the transmission performance of the original radome, resulting in the inability to solve the compatibility of high transmission and out of band stealth through traditional methods. Therefore, there is an urgent need for metasurface designs that combines the functions of enhancing transmission in the wide angle domain within the band and reducing the radar cross-section (RCS) out of the band.

**Figure 1:**
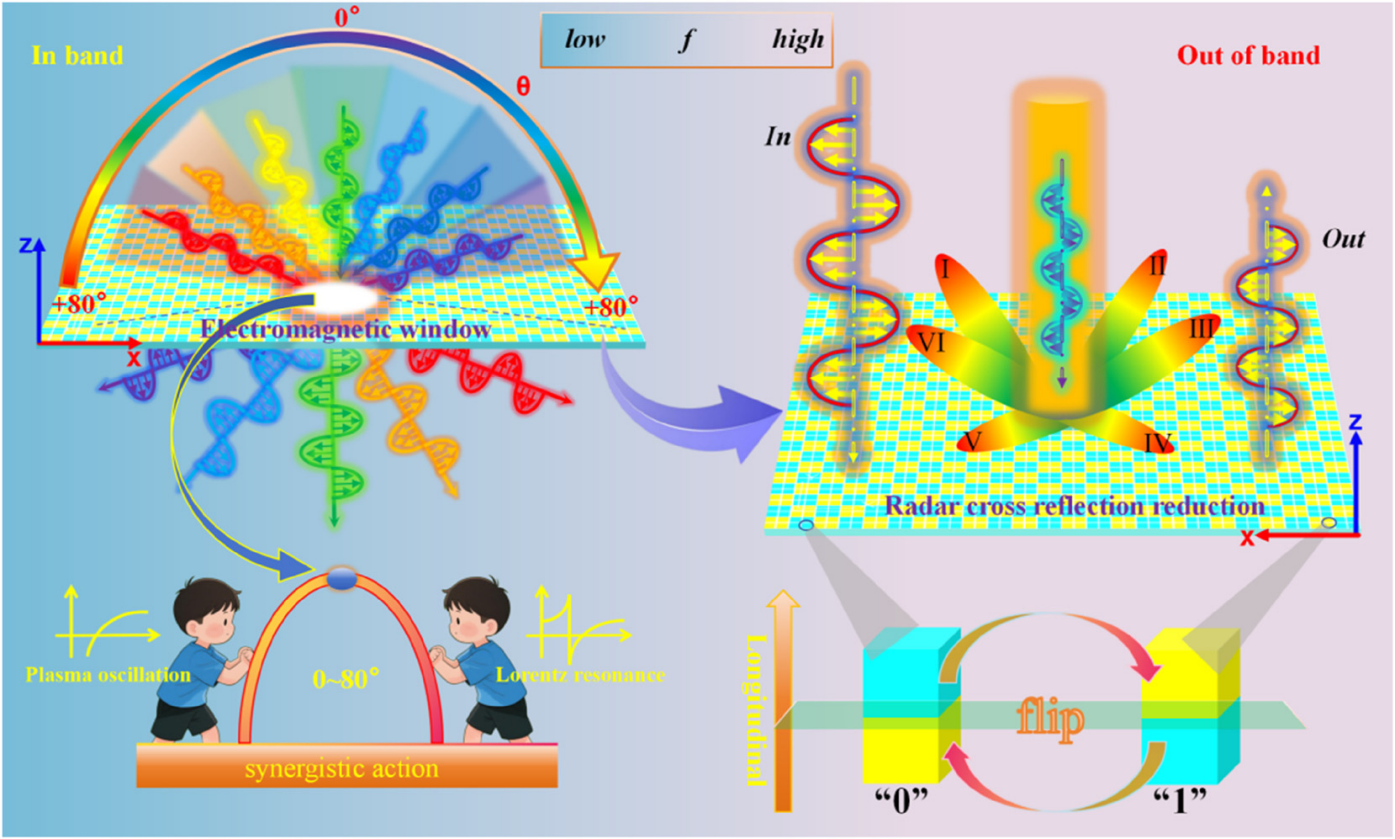
Schematic of this work. The cascading metasurface forms a stable wide-angle-domain electromagnetic window under dual polarization through the synergistic action of the plasma effect of the metal mesh and the Lorentz resonance effect of the metal patch. Its function is further expanded by breaking up the reflected waves through coding, thereby reducing the RCS.

As early as in 2014, Professor Tiejun Cui’s team proposed the concept of coding metasurface, which reduces RCS by using the reflection phase difference between different unit cells to scatter reflected waves in different directions in space [26]. In 2017, they also proposed to encode the metasurface consisting of lossy structures, which further improved the RCS reduction performance of the coding metasurface [27]. With the development of coding metasurfaces, they have special research significance in beam regulation [28], achieving polarization insensitivity [29], and independently controlling the transmitting and receiving channels [30]. On this basis, scholars have gradually realized that, in addition to the control of reflection or radiation characteristics, the coding regulation of transmission waves also holds significant theoretical value and application potential. Later, scholars began to pay attention to the effect of coding on transmitted waves. In 2021, Hongchen Chu’s team proposed a kind of coding metasurface that can realize one-way perspective of human eyes in the visible frequency band based on the principle of reciprocity [31]. In 2022, they demonstrated that the design of metasurfaces based on reciprocity is also applicable to the microwave frequency band [32]. As shown in Figure 1, the multi-layer cascade metasurface is composed of several identical atomic units but with different orientations.

**Figure 2:**
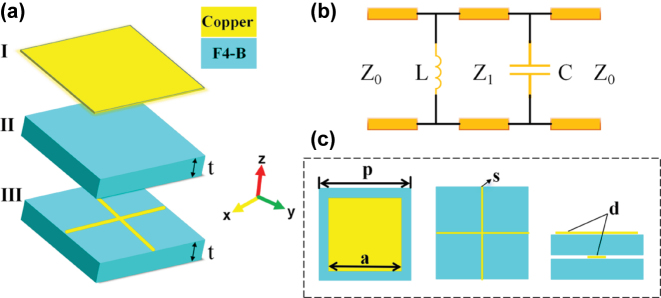
The structural parameters of the cascade metasurface meta-atom. (a) The thickness of the substrates *t* = 4 mm. (b) The equivalent circuit of the cascade metasurface. (c) The period *p* = 12 mm, the line width of the metallic mesh is *s* = 0.05 mm, the side length of a square metallic patch *a* = 11.9 mm, and the thickness d of all metallic structure is 0.02 mm.

Inspired by the above work, different from the traditional methods that often utilize the changes of metamaterial structure parameters in the transverse dimension to regulate electromagnetic characteristics, we propose an electromagnetic window design scheme based on longitudinally flipped encoded metasurfaces. This scheme can simultaneously achieve the reduction of out-of-band RCS and the wide-angle domain angle transmission performance in band. First, we design the initial cascade metasurface with band-pass performance around 1.6 GHz by equivalent circuit analysis. As shown in Figure 2(a), the cascade metasurface is composed of a layer of square metallic patches, a layer of metallic meshes and two poly tetra fluoro ethylene (PTFE) substrates. This asymmetric structure design results in significant changes in the reflection phase of meta-atom before and after flipping, which makes preparation for subsequent coding processing. Subsequently, based on the design principle of coding metasurface, the meta-atoms with the metallic patch on the lower surface is defined as “low” meta-atoms, the ones with the metallic patch on the upper surface is defined as “up” meta-atoms (flip the “low” meta-atom to get the “up” meta-atom), and 4 same meta-atoms arranged by 2 × 2 arrays are defined as a “0” or “1” lattice. Then we use 010101 and 001011 sequences to encode the initial cascade metasurface. Considering the reflection phases of the two distinct meta-atoms exhibit a certain degree of difference, the reflected waves from the cascade metasurface are dispersed in multiple directions after encoding, thereby reducing the RCS. Owing to the principle of reciprocity, the transmission phase of the two meta-atoms remains identical, ensuring that the transmitted wave from the cascade metasurface remains unchanged after encoding, thereby preserving the initial transmission performance. After a series of simulations in CST Microwave Studio, a set of proof-of-principle prototypes were designed, fabricated and measured. The experimental results are almost identical with the simulation results and they all confirm the correctness of our strategy. This research introduces a novel approach for electromagnetic windows that combines the functions of in-band wide-angle domain transmission enhancement and out-of-band radar cross-section (RCS) reduction, which has significant potential application value in various application scenarios.

## 2 Theory and design

The initial cascade metasurface comprises a layer of square metallic patches, a layer of metallic mesh and two PTFE substrates. The meta-atom and the relevant structural parameters are shown in Figure 2(a) and (c), where the relative dielectric constant of the substrate is *ɛ*_*r*_ = 2.65. As shown in Figure 2(b), the cascade metasurface can be equivalent to a parallel LC resonant circuit, in which the gap between the metallic patches can induce the equivalent capacitance, and the metallic mesh forms the equivalent inductance. The free space in the side and two substrates can be equivalent to the resistance, whose impedance is expressed as *Z*_0_ = 377 Ω and $Z_{1}=Z_{0}/{\left(\varepsilon_{r}\right)}^{1/2}$ respectively. To gain a clearer understanding of the relationship between the physical dimensions of the structure and its equivalent capacitance and inductance, numerous research efforts have developed empirical formulas for estimating these equivalent circuit parameters. Relatively accurate approximate expressions for the equivalent inductance and capacitance of metallic unit cells are given as follows Eqs. (1) and (2) [33]:

(1) $$C=\varepsilon_{0}\varepsilon_{r}\frac{2p}{\pi }\ln\left({\left(\sin\frac{\pi \left(p-a\right)}{2p}\right)}^{-1}\right)$$

The capacitance value is determined by the periodic size *p*, the metallic patch size *a* and the equivalent dielectric constant of the substrate *ɛ*_*r*_. Increasing *p* and *a* will increase *C*. The equivalent inductance *L* can be expressed as Eq. (2).

(2) $$L=\mu_{0}\frac{p}{2\pi }\ln\left({\left(\sin\frac{\pi s}{2p}\right)}^{-1}\right)$$

(3) $$C_{m}=\varepsilon_{0}\varepsilon_{r}S/d$$

The equivalent inductance value is determined by the periodic size *p* and the line width of the metallic mesh *s*. Increasing the *p* and decreasing *s* will increase *L*. A certain mutual capacitance exists between the metal meshes and the metal patches. According to the definition of capacitance *C* = *ɛ*_0_ *ɛ*_*r*_ *S*/*d*, the mutual capacitance *C*_*m*_ between the metal patches and the metal meshes can be derived, which primarily depends on the overlapping area of the two metallic structures and the distance *d* between them. Due to the relatively large separation between the metal patches and the metal meshes, the resulting mutual capacitance *C*_*m*_ is small and can be neglected. Therefore, the overall capacitance of the system can be regarded as solely arising from the mutual capacitance *C* between the metal patches. According to the parallel resonant circuit $f_{0}=1/2\pi \sqrt{LC}$ , the resonant frequency *f*_0_ is proportional to *p* and *a*, and inversely proportional to *s*.

**Figure 3:**
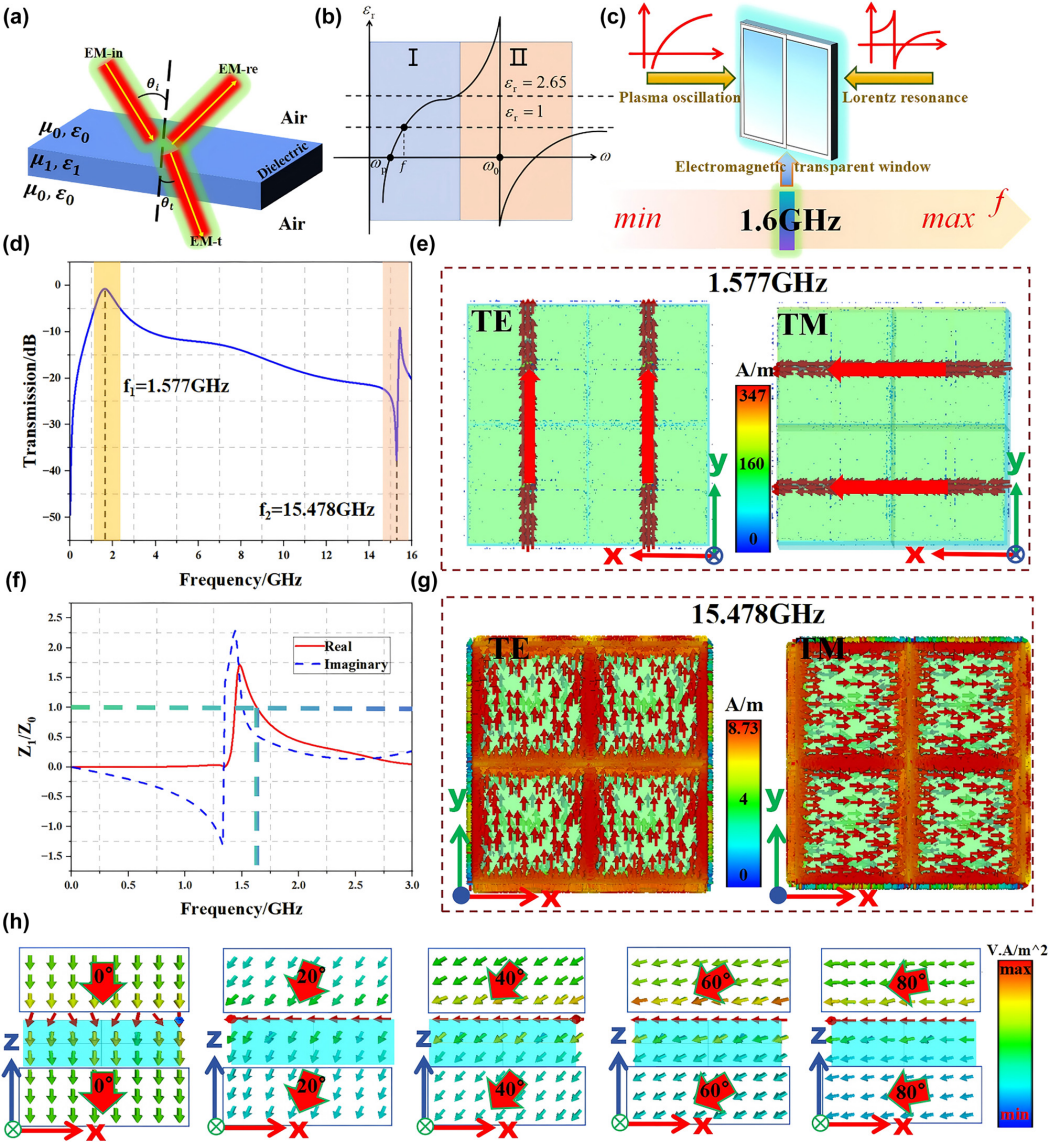
Physical background and the principle of antireflection spectroscopy. (a) A schematic diagram illustrating the reflection of EM waves when they enter a dielectric plate from air. (b) The variation of the relative permittivity of the substrate loaded with metal mesh-like lines and metal patch structures with frequency. (c) Schematic diagram of the principle of forming an electromagnetic transparent window. (d) Transmission spectra after loading both the metal mesh-like lines and the metal patch structures simultaneously. (e) Surface current distribution under TE and TM polarization at 1.577 GHz. (f) The real part and the imaginary part of the relative equivalent impedance of the substrate under TE polarization after loading metal mesh lines and metal patch structures at an incident angle of 0°. (g) Surface current distribution under TE and TM polarization at 15.478 GHz. (h) The energy flow within the substrate when TE or TM polarized waves are incident at five different angles.

According to Snell’s law [34], [35], [36], [37], as shown in Figure 3(a), the transmission efficiency is maximized when the impedance of air matches that of the cascade metasurface. Here, *η*_0_ and *η*_1_ respectively denote the intrinsic wave impedances of air and the cascade metasurface. *θ*_*i*_ and *θ*_*t*_ respectively represent the incident angle and the refraction angle; *μ*_0_ and *ɛ*_0_ represent the magnetic permeability and dielectric constant of air, while *μ*_1_ and *ɛ*_1_, respectively, denote the effective magnetic permeability and effective dielectric constant of the cascade metasurface. $Z_{0}^{TE/TM}$ and $Z_{1}^{TE/TM}$ respectively represent the equivalent wave impedances of air and the cascade metasurface under TE and TM polarizations, and Γ represents the reflection coefficient.

(4) $$\eta_{0}=\sqrt{\frac{\mu_{0}}{\varepsilon_{0}}},\eta_{1}=\sqrt{\frac{\mu_{1}}{\varepsilon_{1}}}$$

(5) $$Z_{0}^{TE}=\frac{\eta_{0}}{\cos\theta_{i}}=\sqrt{\frac{\mu_{0}}{\varepsilon_{0}\left(1-{sin}^{2}\theta_{i}\right)}}$$

(6) $$Z_{1}^{TE}=\frac{\eta_{1}}{\cos\theta_{t}}=\frac{\mu_{1}}{\sqrt{\varepsilon_{1}\mu_{1}-\varepsilon_{0}\mu_{0}{sin}^{2}\theta_{i}}}$$

(7) $$Z_{0}^{TM}=\eta_{0}\cos\theta_{i}=\sqrt{\frac{\mu_{0}\left(1-{sin}^{2}\theta_{i}\right)}{\varepsilon_{0}}}$$

(8) $$Z_{1}^{TM}=\eta_{1}\cos\theta_{t}=\sqrt{\frac{\mu_{1}}{\varepsilon_{1}}\left(1-\frac{\varepsilon_{0}\mu_{0}{sin}^{2}\theta_{i}}{\varepsilon_{1}\mu_{1}}\right)}$$

(9) $$\Gamma =\frac{Z_{1}^{TE/TM}-Z_{0}^{TE/TM}}{Z_{1}^{TE/TM}+Z_{0}^{TE/TM}}$$

When TE or TM waves are incident on the cascade metasurface, the localized electrons within the metal mesh lines will generate plasma oscillations, and the equivalent dielectric constant of the cascade metasurface will change. The metal mesh-like line causes the equivalent dielectric constant of the substrate to vary with frequency due to plasma resonance is shown in Figure 3(b).I, which conforms to the Drude model. The equivalent dielectric constant of the substrate no longer remains constant but increases nonlinearly with frequency (from less than 0 to the dielectric constant of the bare substrate itself). The trend of the equivalent dielectric constant of the substrate with respect to frequency caused by Lorentz resonance of the metal patch is shown in Figure 3(b).II, which conforms to the Lorentz model. Among them, the so-called plasma frequency *ω*_*p*_ represents that the dielectric constant of the substrate as a whole is exactly equal to zero, and the dispersion characteristics of the substrate will change. As shown in Figure 3(c), when metal mesh and metal patch are added to the substrate, the resonant frequency of the metal patch and the plasma frequency of the metal mesh are jointly regulated and acted on the parameter *f* through the coupling effect, so that the wave transmission window is stabilized at *f*.

In order to further analyze the aforementioned coupling effects, we separately analyzed the interaction between the metal meshes and the metal patches. When metal meshes are introduced onto the metal patches, the plasma oscillation induced by the meshes follows the Drude model [38], which reduces the effective permittivity of the substrate at the high-frequency end. As a result, the wavelength corresponding to the Lorentz resonance decreases, leading to a shift of the resonant frequency toward higher values. Similarly, when metal patches are added to the metal meshes, the Lorentz resonance effect introduced by the patches conforms to the Lorentz model [38], which increases the effective permittivity of the substrate at the low-frequency end. This causes an increase in the wavelength corresponding to the plasma frequency, thereby shifting the plasma frequency toward lower values. As the plasma frequency shifts downward, the frequency *f* corresponding to impedance matching also shifts accordingly to a lower frequency. Therefore, it can be concluded that the transmission window observed at frequency *f* results from the combined action of both the metal meshes and the metal patches.

At this time, the overall equivalent dielectric constant of the cascaded metasurface is exactly one. As shown in Figure 3(f), at this time, a good impedance matching is formed between the cascade metasurface and the air on 1.6 GHz. According to formula (3)–(7), we can obtain: $Z_{0}^{TE}$ *=* $Z_{1}^{TE}$ , $Z_{0}^{TM}$ *=* $Z_{1}^{TM}$ . Then, based on formula (8), the reflection coefficient is calculated to be zero, and the reflection rate is reduced. According to the law of conservation of energy, the transmittance is enhanced.

As shown in Figure 3(d), a transmission channel is formed not only at 1.6 GHz but also at 15.47 GHz, although with weaker transmission strength. This channel corresponds to the Lorentz resonance mode of the metasurface unit structure. Unlike the dominant mode responsible for transmission at 1.57 GHz, the Lorentz resonance mode typically exhibits more complex field distributions and distinct characteristics. The operating frequency of the Lorentz resonance mode (15.47 GHz) is much higher than that of the mode at 1.57 GHz. According to the skin effect theory, the skin depth $\delta =\sqrt{2/\left(2\mathrm{\pi f}\mu \sigma \right)}$ is inversely proportional to the square root of the frequency. The nearly tenfold increase in frequency leads to a significantly reduced skin depth and increased current density, thereby considerably enhancing ohmic losses on the metal surface and consequently reducing transmission efficiency. This is also the reason why only the transmission peak at 1.57 GHz is considered subsequently.

To further analyze the frequency selection principle of cascade metasurface, as shown in Figure 3(d), we selected two frequency points of 1.577 GHz and 15.478 GHz as the monitoring objects. The surface currents of TE and TM polarized waves at the incident angle of 0° were observed in CST. According to Figure 3(e) and (g), it can be seen that there are strong surface currents on the metal mesh-like line structure at low frequencies, while the currents on the metal patch is relatively weak. This indicates that at the low-frequency end, the plasma resonance effect of the metal mesh-like line structure plays a role in regulating the frequency point *f*. At the high-frequency region, the metal patch has a relatively strong surface current, while the current on the metal meshes are relatively weak. This indicates that at the high-frequency end, the Lorenz resonance effect of the metal patch plays a role in regulating the frequency point *f*. Therefore, metal meshes and metal patch structures can be utilized to enhance the impedance matching of cascade metasurface at the frequency f, thereby enabling the realization of the transmission characteristics at this frequency point. Furthermore, we continue to analyze the energy flow distribution of the upper and lower surfaces of cascade metasurface at frequency *f* based on the 0° reference point. Measurements are taken every 20°. As can be seen from Figure 3(h), when the EM waves passes through the cascade metasurface, there is no obvious deflection on the upper and lower surfaces of the cascade metasurface. Moreover, it passes through the substrate along a smooth path (the refractive angle is almost equal to the incident angle), and the energy flow magnitudes on both sides of the cascade metasurface are basically the same. This indicates that the impedance matching between the cascade metasurface and the air is good, and the EM waves transmission rate is high.

**Figure 4:**
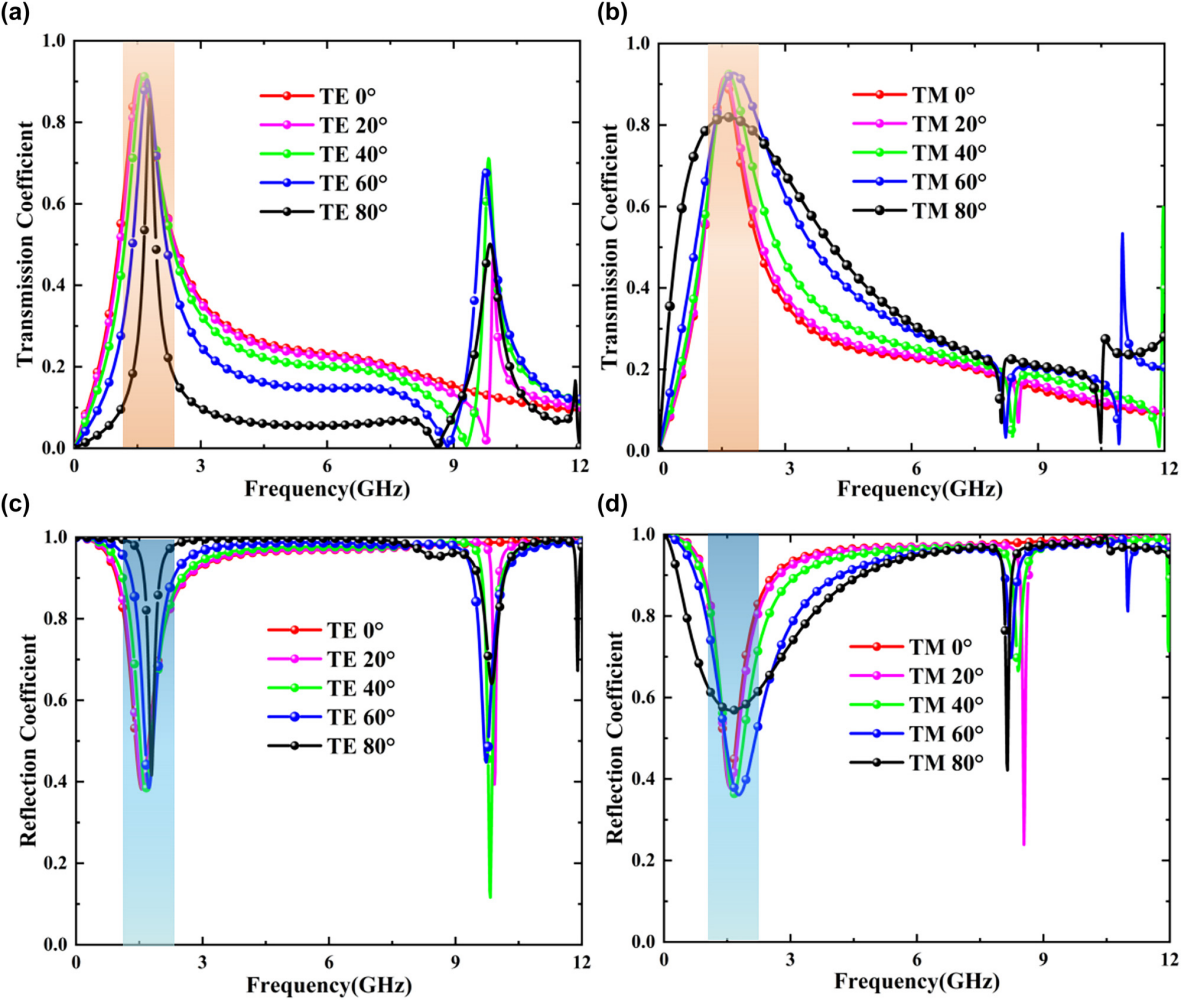
Transmission and reflection spectra under dual polarization. (a) and (b) The transmission coefficient of the EM waves under TE-polarization and TM-polarization. (c) and (d) The reflection coefficient of the EM waves under TE-and TM-polarization.

According to this rule, relevant dimension parameters are adjusted, and the “unit cell” boundary and frequency domain solver of CST microwave studio is used for simulation calculation, and the results obtained are shown in Figure 4. It can be observed that the initial cascade metasurface exhibits excellent band-pass performance for both TE- and TM-polarized electromagnetic (EM) waves around 1.6 GHz. However, nearly all EM waves outside the passband are reflected. Therefore, although the transmission performance of cascade metasurface is very good, it has a large out-of-band RCS.

**Figure 5:**
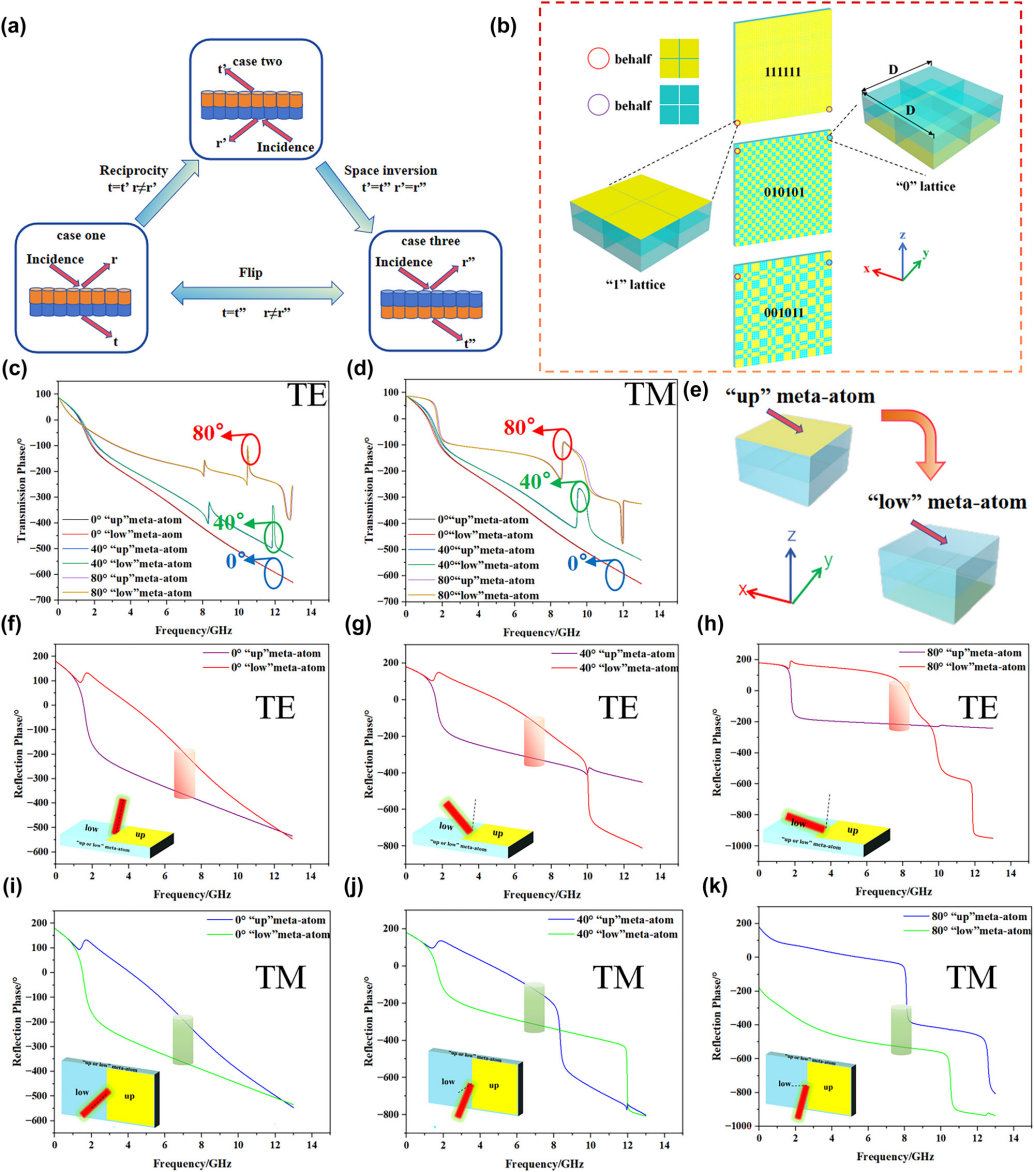
The underlying mechanism of the reciprocity theorem and its specific application to the subscript form of coding. (a) The reciprocity principle and space-inversion invariance jointly guarantee the unchanged transmission under the flip operation for the same incidence while the reflections can be distinctly different, r and t respectively represent the reflection waves and the transmission waves. (b) The “0” lattice and “1” lattice with sides of length *D* = 24 mm, and the flip coded cascade metasurface with the periodic sequence of 111111, 010101 and 001011. (c) and (d) Transmission phases of TE/TM polarization at 0°, 40° and 80°. (e) The two types of meta-atoms are related to each other by the space-inversion (flip) operation. (f), (g) and (h) Reflection phases of TE polarization at 0°, 40° and 80°. (i), (j) and (k) Reflection phases of TM polarization at 0°, 40° and 80°.

With a high-performing initial cascade metasurface in place, we commence to flip coding it. As shown in Figure 5(e), we define “low” meta-atom for the metallic patch on the lower surface of the substrate and “up” meta-atom for the metallic patch on the upper surface of the substrate, the “up” meta-atom can be regarded as the “low” meta-atom flipped 180°. Then, in CST, we incident TE-polarized waves along the negative direction of *z-*axis, and simulated the cascade metasurface formed by the two-dimensional periodic extension of two different meta-atoms. As shown in Figure 5, it is evident that the reflection phase of the “low” meta-atom and the “up” meta-atom differs due to the distinct positions of the metallic patch. However, the transmission phase of both meta-atoms remains identical.

As shown in Figure 5(a) case one, when an EM wave is incident from the top onto a plate composed of up-down asymmetric elements, Snell’s law dictates that, in the absence of energy loss, a portion of the wave is reflected while the remainder is transmitted. When the electromagnetic wave is incident from the bottom of the plate at the same angle (as shown in Figure 5(a) case two, the phase of the reflected wave changes, whereas the phase of the transmitted wave remains unchanged). Consequently, *r* ≠ *r*′, while *t* = *t*′. Similarly, as shown in Figure 5(a) case three, if the wave remains incident from the top of the plate but the plate is rotated by 180°, the phase of the reflected wave will exhibit the same change, whereas the phase of the transmitted wave will remain unchanged. Consequently, *r* ≠ *r*″, and *t* = *t*″. When the spatial configuration and direction are reversed, as shown in Figure 5(c) and (d), the transmission phases of the three characteristic angles remain basically unchanged under TE and TM polarization, which provides a basis for maintaining the stability of the transmission effect by using the reciprocity theorem in the subsequent process. As shown in Figure 5(f)–(h), under TE polarization, the reflection phases of the three characteristic angles differ by 180° near 7 GHz. As shown in Figure 5(i)–(k), under TM polarization, the reflection phases of the three characteristic angles also differ by 180° near 7 GHz. This is for the subsequent dispersion of the reflected waves using the differences in the reflection phases. Moreover, the variation laws governing the reflection phase and the incident phase differ. This phenomenon is consistent with the reciprocity principle [39], [40], which forms the theoretical foundation for our subsequent encoding of cascade metasurface.

The main function of coding metasurface is to disperse the reflected EM wave to different directions in space to achieve RCS reduction, requiring a key element which must have a reflection phase difference [26], [27]. As shown in Figure 5(f)–(k), we have verified that the reflection phase of “low” meta-atom and “up” meta-atom has a difference, and the difference is exactly 180° near 7 GHz, which fully meets the requirements of coding metasurface. And as shown in Figure 5(a), we also verify that the reciprocity principle is true for the initial cascade metasurface, which means that ideally if we code the initial cascade metasurface with “low” meta-atom and “up” meta-atom as the encoding elements, we can not only break the reflected wave but also keep the transmitted wave unchanged. It not only maintains the transmission performance of cascade metasurface but also reduces its RCS.

As shown in Figure 5(b), according to the design criteria of coding metasurface [41], we arrange 4 “low” meta-atoms in a 2 × 2 array to form a “0” lattice and arrange 4 “up” meta-atom in the same way to form a “1” lattice. The side length of the lattices *D* = 24 mm. We then arrange the designed lattice in particular periodic sequences to form the flip coding cascade metasurface. Following the above, we now discuss the strengths of the different encoding approaches in Figure 5(b). The 010101 and 001011 coding sequences produce significantly different scattering patterns. We define “0” to represent a phase of 0° and “1” to represent a phase of 180° after longitudinal flipping. Due to the 180° phase difference between the two differently arranged unit types, destructive interference occurs across the surface structure, thereby weakening the reflected wave. From the perspective of the law of energy conservation, the cancelled energy is not lost but is redistributed, forming weaker, more dispersed scattering lobes. As shown in Figure 7(b) for the 111111 coding sequence, all units have identical phases. Consequently, no mutual interference occurs among the reflected waves, resulting in a far-field pattern where energy is highly concentrated in the direction of the incident electromagnetic wave. In contrast, the 010101 coding sequence shown in Figure 7(b), while still a periodically arranged structure, introduces a 180° phase-flipped unit adjacent to each original unit. The far-field simulation reveals the presence of relatively strong sidelobes in its radiation pattern, while a significant reduction in RCS is achieved in the specular direction. Regarding the 001011 sequence in Figure 7(b), this coding sequence is introduced to design an unconventional, aperiodic arrangement that incorporates randomness. By designing such a sequence for effective comparison with the two aforementioned types, its far-field pattern demonstrates that the scattered energy is dispersed into various directions in space, achieving a distribution of scattering energy that is as flat and uniform as possible.

## 3 Simulation results

**Figure 6:**
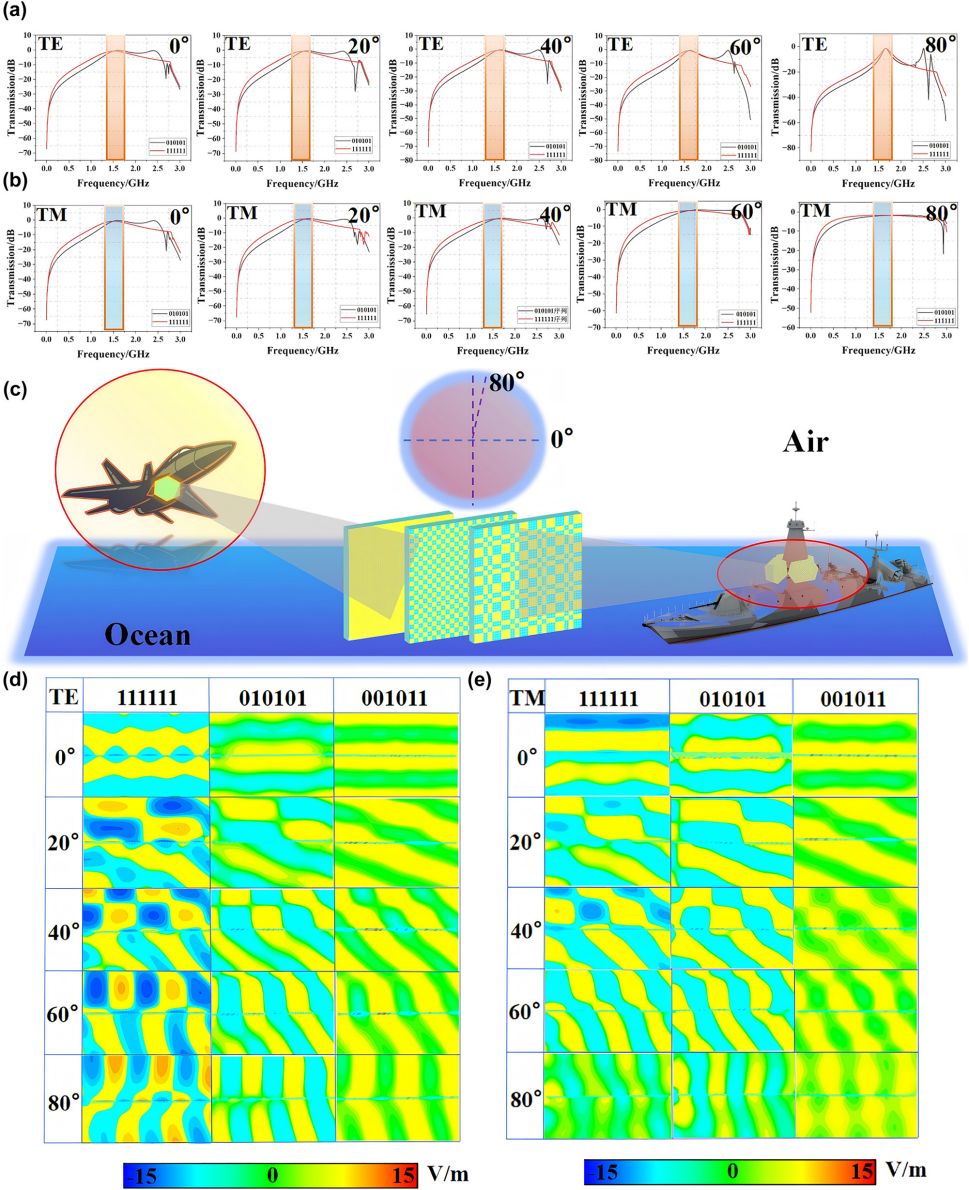
Verification and test results of the anti-reflection principle of three sequences at all angles under dual polarization. (a) and (b) Under TE or TM polarization, the transmittance of 111111 and 010101 sequences corresponds to the incidence angle of 0°–80°. (c) Schematic diagram of the incident angle of the flat antenna radome on the simulated ship or aircraft. (d) and (e) Electric field distribution of three sequences 111111, 010101 and 001011 under TE or TM polarization.

In CST, we test the transmittance of two sequences 111111 and 010101 under different polarizations through frequency domain solver, and set the incidence angles to 0°, 20°, 40°, 60° and 80°, so as to simulate the actual situation of various incidence angles. The simulation test diagram is shown in Figure 6(a) and (b). We use −1 dB as the standard to divide the bandwidth, and it can be seen from the figure that the best transmission effect is concentrated around 1.6 GHz. In order to further observe the transmission effect of 001011 sequence at 1.6 GHz, we used time domain solver to conduct electric field simulation test, and compared the results with the two sequences 111111 and 010101. The results are shown in Figure 6(d) and (e). It can be seen from the figure that EM waves can pass almost without loss through the metasurface of the three coding sequences. According to the above simulation results, it can be seen that when electromagnetic waves are incident at a large incident angle under TM polarization, their high selectivity is somewhat lacking. The reason for this is that under TM polarization, the Brewster effect exists, which causes the reflected waves to disappear, thus achieving complete transmission.

**Figure 7:**
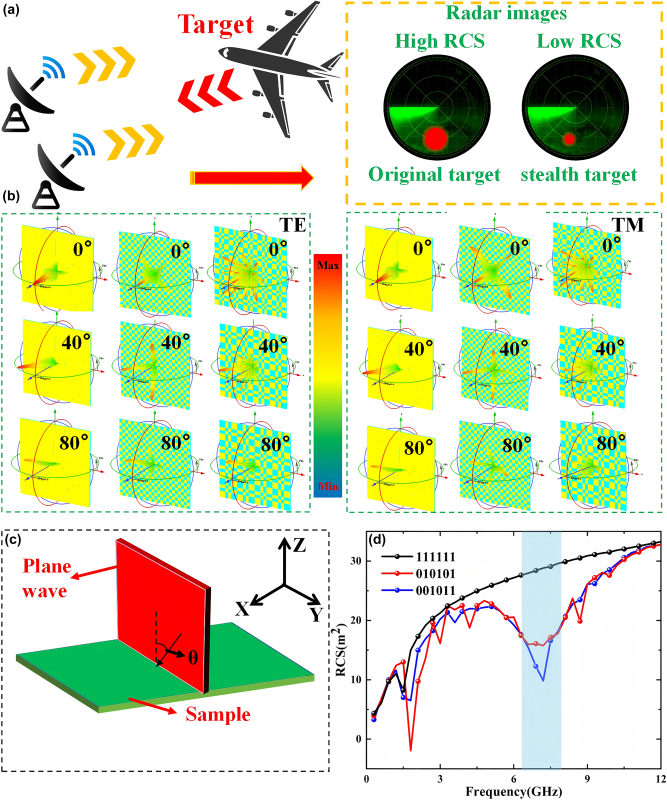
The effect diagram of RCS reduction and the far-field simulation test diagram. (a) Schematic representation of the interaction between simulated radar-emitted EM waves and the target. (b) Far-field simulation results for the three sequences 1111110, 10101, and 001011 under TE or TM polarization with incident angles ranging from 0° to 80°. (c) Far-field measurement models for the three sequence samples were conducted using plane wave illumination. (d) Linear plots depicting the RCS characterization of the three sequences at a 0-degree incidence angle.

In CST, we modeled the flip coding cascade metasurface with the periodic sequences of 111111, 010101 and 001011, all of the dimensions of 576 mm × 576 mm (the initial cascade metasurface can be regarded as the flip coding cascade metasurface with the periodic sequence of 111111). We configured the plane wave in the direction of electric field always along the *y*-axis normally incident on the established cascade metasurface along the negative direction of the *z*-axis, and the boundary conditions in the three directions were set as “open add space”. Subsequently, as shown in Figure 7(c), the time-domain solver was employed to solve the problem, and the RCS values of the three cascade metasurface structures were obtained, as shown in Figure 7(d). It can be clearly seen that compared with the initial cascade metasurface, the RCS of the coding cascade metasurface around 7 GHz is significantly reduced, but the bandwidth is relatively narrow. To expand the bandwidth, we can introduce absorbing materials on the basis of the above structure, thereby achieving a reduction in broadband RCS. On the basis of maintaining the reduction of broadband RCS, it is more meaningful to achieve a transmission band with stable angles. Although this article has achieved in-band transmission enhancement and out-of-band RCS reduction efficiency, there is still some room for improvement. While ensuring the stability of the transmission channel angle, achieving a wider RCS reduction has a better application space for practical effects. Therefore, we have concluded that on the basis of this article, appropriate absorbing materials can be added to achieve a wider RCS reduction without affecting the efficient transmission within the band.

In order to further verify our design scheme and confirm that the transmitted wave of cascade metasurface has not changed after coding, we respectively monitored the far-field energy distribution of cascade metasurface at 7 GHz, and the results are shown in Figure 7(b). It can be clearly seen that compared with the initial cascade metasurface, the reflected waves of the flipped coding cascade metasurface are dispersed in different directions.

## 4 Experiment

**Figure 8:**
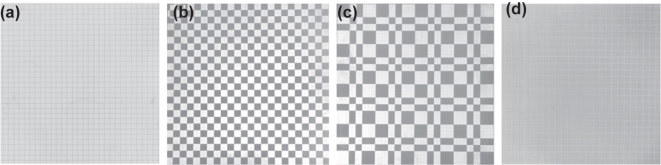
Experimental test substrates for three sequences. (a), (b) and (c) Metallic patch arranged by sequences of 111111, 010101, and 001011 are etched on one side of the PTEF plate. (d) Etch the metallic mesh on the other side of the PTEF plate.

To verify our design scheme we fabricated a set of prototypes and measured them. As shown in Figure 8 we fabricated 4 mm thick PTFE plates with size of 576 mm × 576 mm and etched metallic meshes or metallic patch arranged according to different coding sequences on its surface with printed circuit technology (PCT). The structural parameters of the patch and meshes were the same as those in Figure 2(a). As shown in Figure 8, two plates were assembled to reconstruct the designed cascade metasurface structures. The transmission and reflection characteristics of the prototype were measured separately by using horn antennas within an electromagnetic anechoic chamber and an arched frame.

**Figure 9:**
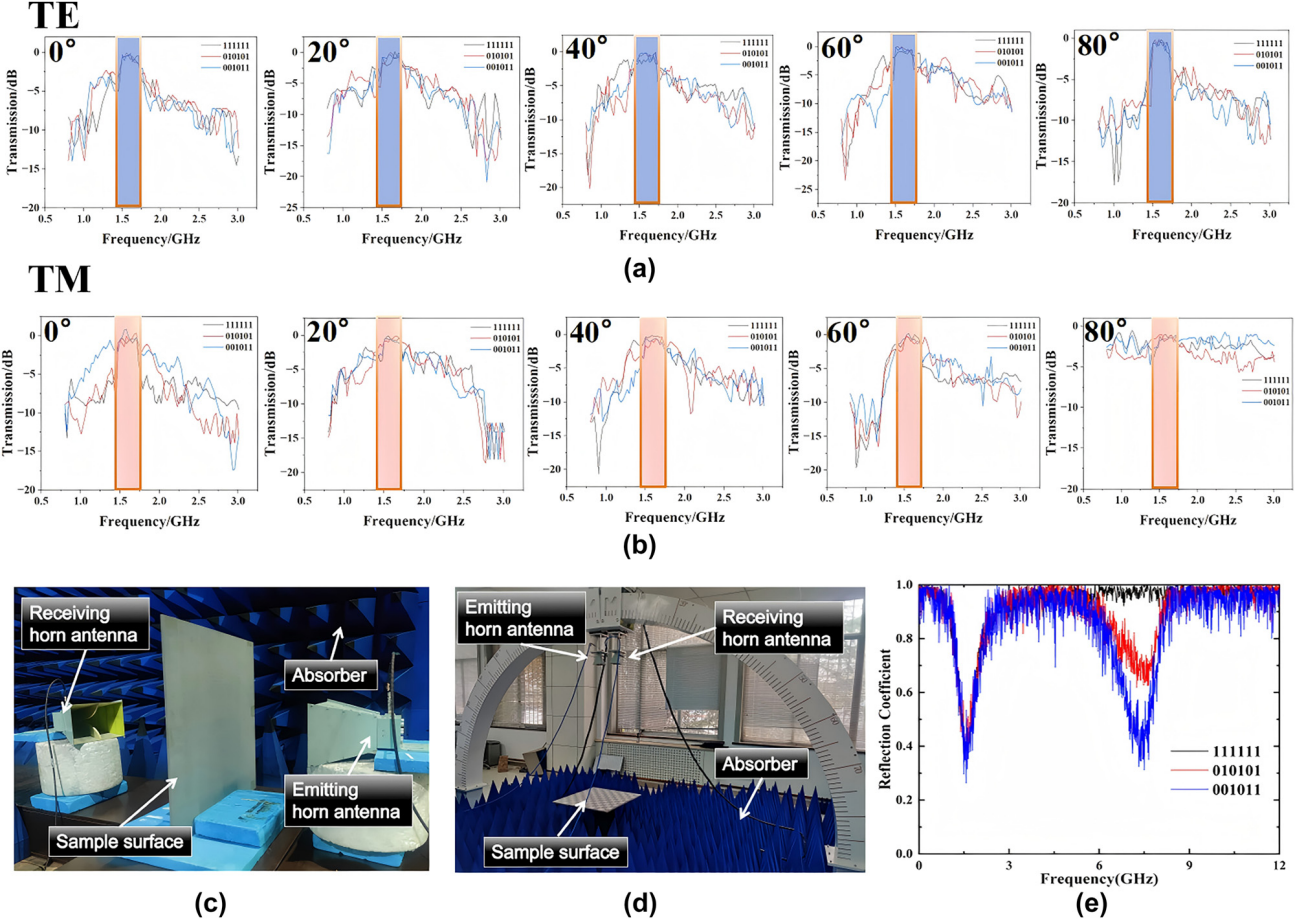
Schematic diagram of the transmission and RCS experimental test results of the three sequences under dual polarization. (a) Under TE polarization, 0°–80° 111111, 010101 and 001011 transmission lines of three coding sequences. (b) Under TM polarization, 0°–80° 111111, 010101 and 001011 transmission lines of three coding sequences. (c) Measured the transmission of the prototype in an EM dark room. (d) Measured the reflection of the prototype in an arched frame. (e) The reflection coefficient of coding cascade metasurfaces with periodic sequences of 111111, 010101, and 001011.

To ensure the accuracy of the experimental test, each time we measure the wave transmission test at different angles under different polarizations, we will perform calibration and normalization tests to avoid accidental errors. Secondly, for each measurement result, we conducted multiple repeated experiments. Moreover, our testing environment is carried out in a microwave anechoic chamber. The absorbing sponge in the anechoic chamber can absorb most of the clutter signals, effectively preventing these signals from affecting the experimental tests. Measured results are shown in Figure 9. Compared with 111111 sequence, flip coding cascade metasurfaces with 010101 or 001011 sequence has significantly lower reflection but basically the same transmission. Since the physical significance of RCS and reflection is basically the same at 0° incident angle, the decrease of reflection can indirectly indicate the decrease of RCS. Our measured results deviate from the ideal situation mainly because there are some errors in the fabricating and there is also a small amount of clutter in the dark room. This causes a certain degree of jitters in the test results, but the above-mentioned jitters noise can be effectively filtered by adding a time domain gate. Even so, the measured results still prove the correctness and performance of our scheme.

## 5 Conclusions

This paper presents a design scheme for an electromagnetic transparent window based on the flipping of coding element surfaces. This approach enables the simultaneous achievement of stable wave transmission across wide angles within the band and the reduction of radar cross-section (RCS) out of the band. First, the initial band-pass cascade element surface is designed. Subsequently, the cascade element surface is flipped and encoded by using periodic sequences of “010101” and “001011”. Owing to the protection provided by the reciprocity principle, the reflected wave is scattered while the transmitted wave remains unaffected. This not only effectively reduces the radar cross-section (RCS) but also preserves the original transmission performance. Thus, the simultaneous resolution of compatibility issues between electromagnetic communication and electromagnetic stealth has been achieved through the longitudinal freedom design of a single structure. To validate our design scheme, we conducted simulation experiments by using CST to obtain the RCS of cascade metasurfaces with different coding sequences and monitored their far-field energy distribution at 7 GHz. A series of proof-of-principle prototypes were designed, fabricated, and measured to validate this scheme. The measured results are in agreement with the simulation results, thereby validating both the correctness and performance of this scheme. This work introduces a novel approach to designing metasurface with low RCS, which holds significant. This work paves a new route to multi-functional EM windows and may find wide applications in new-generation communication, radar systems and others.
